# Changing Infrastructural Practices: Routine and Reproducibility in Automated Interdisciplinary Bioscience

**DOI:** 10.1177/0162243919893757

**Published:** 2019-12-09

**Authors:** Robert Meckin

**Affiliations:** 1 Sociology, University of Manchester; 2 The Cathie Marsh Institute, University of Manchester; 3 The Morgan Centre for Research into Everyday Lives, University of Manchester

**Keywords:** infrastructure, engineering biology, automation, reproducibility, practices, synthetic biology

## Abstract

Proponents of engineering and design approaches to biology aim to make interdisciplinary bioscience research faster and more reproducible. This paper outlines and deploys a practice-based approach to analyses of infrastructure that focuses on the routine epistemic activities and charts how two such routines are unsettled and resettled in the background of epistemic culture. This paper describes attempts to bring about new research infrastructures in synthetic biology using robotics and software-enabled design. A focus on the skills of pipetting shows how established manual labor has to be reconfigured to fit with novel robotic automations. An analysis of curating frozen materials shows that automated design presents new problems for the established activities of storing and retrieving biological materials. These movements, while transient, have implications for organizing interdisciplinary collaboration, research productivity, and enabling greater reproducibility. This paper explores the idea of infrastructure as practice and shows how this has important implications for studies of research infrastructures. This article discusses the main contributions of this approach for analysts of infrastructure in terms of movements, temporalities, and ethics and offers suggestions for what the research implies for synthetic biology.

## Introduction

New research infrastructures are changing how knowledge is produced. Biological science is being restructured in particular ways to realize epistemological and economic goals, particularly in the field of synthetic biology—an engineering-design approach to life. New automated systems are hoped to bring about changes in the productivity of research, including faster, more reproducible, and cheaper approaches to designing biology (Synthetic Biology Leadership Council 2016). These exciting (for some synthetic biologists) developments, where academic groups purchase “flagship” robotic platforms and develop machine-learning applications for designing DNA and optimizing experiments, are still in the process of being integrated into research. Automations are finding their places amid the existing epistemic infrastructures, including the apparatus, laboratories, buildings, and epistemic protocols, of molecular biology and microbiology. This paper argues that bringing new automations and robotics into research can affect and reconfigure existing infrastructural practices in knowledge production.

New configurations of life science research have implications for the tasks researchers do, how they do them, and how they share data and materials (Keating, Limoges, and Cambrosio 1999; Hilgartner 2004; Roosth 2017). Recently funded synthetic biology centers and closely related “engineering biology” research institutions, including biotech companies rich in venture capital, are buying and developing automated design systems and robotic platforms (e.g., Carbonell et al. 2018). The Hamilton Microlab STAR, for example, is a widely used automated liquid-handling machine. One can find the same “Hamiltons” in an academic institution in the North of the UK as in industry-leader Ginkgo Bioworks in Boston, MA. Robotic setups are notably conspicuous as, physically, they take up a space on laboratory benches where perhaps three researchers would have experimented. In comparison, novel design software resides largely on existing computing hardware, although the increase in the complexity of designs and the size of experiments requires increases in computational power and data storage. At various sites, “off-the-shelf” automations are coupled together to produce new infrastructures at a nexus of practices including computer programming, robotics development, research translation, government allocation, and engineering biology.

Over the last two decades, a predominant starting point for science and technology studies (STS) analyses of infrastructure is to conceptualize infrastructures as relational phenomena. The central constructionist and visual analytical metaphors of this relational approach, including “built on an installed base,” “embeddedness,” “embodiment of standards,” “invisibility,” and “transparency” (Star and Ruhleder 1996), have echoed their empirical objects by gaining remarkable stability. To add to these debates, this article takes a position that research infrastructure can be understood as relational by developing further the concept of infrastructure as practice. This paper shows that understanding infrastructure from this basis produces different concerns, questions, and fates for epistemic infrastructures.

The next section explains the importance of an infrastructure analysis to synthetic biology and, more broadly, highlights the key conceptual approaches of the literature on infrastructures and the position taken in this paper. Then, following a short section regarding the case study and methods, the empirical section contains two ethnographic “installments” that highlight how robotic and digital automations are brought to bear on the existing mundane practices in synthetic biology. The first account, focusing on the experimental practice of pipetting, shows how the understandings, material procedures, and engagements are partially reconfigured to accommodate liquid-handling robots. The second account, focusing on the practice of curating frozen biological parts, explores how practices can affect one another such that automated design presents challenges to a crucial element of performing reproducibility in bioscience research. The discussion section draws out implications of this understanding of automated bioscience and how this framing emphasizes particular considerations for analyses of infrastructures, including perspectives on the temporalities, dynamics, and ethics of infrastructures.

## Synthetic Biology Infrastructures

Synthetic biology, an engineering approach to biology, can be seen as a concerted effort to reconfigure the conventions of an incumbent molecular biology using design principles such as standardization and modularization in the hope of “making biology easier to engineer” (Silver 2009). Proponents of synthetic biology have previously instituted a range of conceptual and social apparatuses to structure the field, but the design principles are not stabilized or uniformly enacted (Calvert 2013). Such attempts at “infrastructuring” knowledge production in synthetic biology can meet with resistances that are epistemological, economic, and ethical (Frow 2013). This means that synthetic biology infrastructures emerge amid various (dis)agreements, compromises, and “changing practices” (Balmer, Bulpin, and Molyneux-Hodgson 2016), and the explicit attempts to create general standards for DNA assembly have been realized in local, contingent ways. At the same time, in industrial settings, automations appear to have pushed bioscientists out of the laboratory and into middle management (Roosth 2017). Amid these promises and disruptions, the concepts and procedures developed by synthetic biologists can move between being symbolic of the potential of a synthetic biology approach (e.g., demonstrating biological oscillators as a form of control) to being infrastructures in a particular body of research (e.g., where researchers use biological oscillators in further experiments) or vice versa (Mackenzie 2013). This is important as the elements of a design approach to biology, such as particular automations, become integrated into research at different rates with implications for how research trajectories develop.

Research in STS and cognate literatures that focus on infrastructure consists of three broad and overlapping strands of investigation centered on information infrastructures (Bowker et al. 2010; Ford and Wajcman 2017; Frohlich 2017; Karasti et al. 2016; Plantin et al. 2016), material technologies and buildings (Pinch 2010; Shove, Watson, and Spurling 2015; Street 2012, 2016; Vertesi 2014), and research infrastructures (Hine 2006; Ribes and Polk 2015; Star and Griesemer 1989). Analytically, the articles dealing with information and knowledge production can be divided into studies that focus on “infrastructuring” including assembling, designating, and maintaining infrastructures (e.g., Blok, Nakazora, and Winthereik 2016; Bowker and Star 1999; Parmiggiani and Montiero 2016; Ribes and Polk 2015); studies that examine infrastructure-in-action (e.g., Ford and Wajcman 2017; Merz 2006; Vertesi 2014); and those that do both (e.g., Frohlich 2017). Within these bodies of work, practices tend to be related to infrastructures in two main ways.

Firstly, Star and Ruhleder (1996, 113) argue that infrastructure has “links with conventions of practice” suggesting infrastructure and practice can be thought of as separate entities. An example of this view shows that actors coordinate various infrastructures to communicate, so technologies such as mobile telephones, texting, and teleconferencing software are “aligned” in practice, meaning that communicants do not need to be copresent, which facilitates distributed collaboration (Vertesi 2014). Failure to align these infrastructures successfully means a person can “lose their voice” during online discussions. These types of studies show that infrastructures have political consequences for actors, particularly those who do not (or cannot) access or manage them effectively. The findings are important because they demonstrate the complexity of knowledge production and show how particular infrastructures are interpreted and used in different locations and communities but at the same time keep practice and infrastructure separated.

Alternatively, practices are often addressed as “work practices” such that one person’s actions are another’s infrastructure, for example, the railway engineer’s maintenance work to the commuter’s journey (Bowker and Star 1999; Bowker et al. 2010; Keating, Limoges, and Cambrosio 1999). Discussions about the production of classifications and standards in *Sorting Things Out* (Bowker and Star 1999) show that, for example, the practice of classifying nursing into a range of activities threatens the professional autonomy of nurses. Here, then, the nursing classification “is an actively developing infrastructure” that affects work practices (Bowker and Star 1999, 239). In this reasoning, the practices of classification and standardization become key to developing an infrastructure but are not themselves considered infrastructures nor are the nurses’ activities. However, embedded in this argument is an aside about changes in office work, where the skills of copy typing are claimed to be affected by new managerial strategies and technologies, meaning that typing was largely transferred from secretaries to professionals (Bowker and Star 1999, 239). This idea, of typing as an infrastructural component of office work, is not developed in the book but is the point of departure for the analysis in this paper. Thus, while some of the important literature does talk of practices, the definitions of practice can be limited or not explicitly specified, meaning the literature tends not to consider infrastructure as practice in a systematic way.

Returning to the practices of bioscience, molecular biology has been targeted for automation before. Indeed, during the human genome project, the automated DNA sequencer involved the reorganization of experimental protocols and the “complete substitution of one process by another” (Keating, Limoges, and Cambrosio 1999, 138). However, approaching research infrastructure from the point of view of novel robotic and software automations is at odds with approaches to infrastructure that recognize high-technology environments are “profoundly impacted by the relatively unstudied infrastructure that permeates all its functions” (Star 2002, 117). Analyses emphasize mundane, invisible, or boring objects that are typically in the background (Bowker and Star 1999; Star 1999; Star and Ruhleder 1996). Often, such objects are part of the hidden work of technicians (Shapin 1989). One methodological strategy highlighting these dependencies is to perform an “infrastructural inversion” that makes background structures visible and to emphasize the contingencies in a history of their emergence (Bowker 1994). To study robotics and novel software applications, then, one instead might seek partly to describe the “installed base” (Star and Ruhleder 1996) of molecular biology.

In order to highlight a different set of concerns for analyses of infrastructures in knowledge production, this article concentrates on developing the version of infrastructure as relations between practices and begins formally with the concept of infrastructure as practice. This will be achieved by drawing on a conceptual framework of practices to show the analytical implications for understanding research infrastructure as the relations between epistemic activities. The article advocates an approach that supplements attention to the mundane with STS literature on practices (e.g., Knorr-Cetina 2005; Pickering 1995), philosophical writings (Reckwitz 2002; Schatzki 2002), and studies of consumption (e.g., Warde 2005). On this view, practices are understood as composed of various material and mental actions, understandings, procedural know-how, objects, and affective engagements (the goals, aims, and emotional repertoires constituting a particular practice). Specific practices can be embedded in one another, bundled together in different arrangements (Collins and Kusch 1998; Schatzki 2002), and, as such, can range from the simple to the highly complex depending on the particular cuts an analyst makes.

From this perspective, this paper asks three main questions: What might practice accounts of research infrastructure look like? What happens when infrastructural practices are troubled? What does a formalized account of epistemic practice offer to studies of infrastructure? This paper demonstrates that paying serious attention to relations between practices produces important insights into how accomplishing automated bioscience reconfigures epistemic routines and shows how practices make one another infrastructure.

## Methods and Case

The data for this paper were generated using ethnographic methods including participant observation and interviews over a two-year period as part of a larger project exploring various topics including governance, innovation, and public involvement in synthetic biology. The project involved site visits to various UK synthetic biology research centers and laboratories as well as following actors at conferences and gathering documentary evidence. The data used here consisted of field notes, photographs, and transcribed audio and video recordings. These data were written up as vignettes, read by research participants, and revised in light of their comments, which were predominantly of a technical nature, for example, correcting the terms used for actions of pipetting. The findings and analyses were also iteratively checked with members of my wider research group.

The research center that hosted the main observations was structured into two main groups: a management group of senior scientists, including researchers acting as primary and coinvestigators, and an experimental team of researchers who were colocated in an open plan office. The experimental team was subdivided into three more teams: the design team, the build team, and the test team, each with specialist technical support. The three strong design team constituted computer scientists with backgrounds in control systems engineering and bioinformatics. The five members of the build team all had microbiology backgrounds, with specialisms in enzymes, metabolic pathways, and whole-cell biology. The test team was originally made up of five analytical chemists with expertise in various forms of mass spectrometry for detecting and quantifying chemicals, though one unexpectedly left the group. The laboratories for molecular biology and analytical chemistry were across a gangway with most of the center’s apparatus—several liquid-handling robots and mass spectrometers—positioned on five adjacent benches. The center also had a data team, who provided support for storing and transferring data, and a group of social scientists who supported consideration of social, political, and governance matters.

The theme of infrastructure emerged partly because the researchers in this center, as well as in other synthetic biology endeavors, themselves refer to the creation of new automated infrastructures. The center’s aim was to create a “pipeline” to enable faster creation of microorganisms capable of producing various chemicals, proteins, and materials. The idea was that this pipeline would become a facility for synthetic biology, microbiology, and chemical engineering communities to access in order to develop (usually) *E. coli* strains that produce desired products. They set out several working goals such as the system being “agnostic” to the chemical target and eliminating human error. To this end, the center purchased, developed, and made use of various automations, including robots and machine learning programs, to optimize and speed up experimental design, laboratory processes, and chemical analyses. At the same time, the cost of commercial DNA synthesis has decreased, so the center designs DNA in-house, then orders DNA from companies that make up the DNA sequences, and send the orders back, usually several weeks later.

In this way, the empirical theme of research infrastructure became a significant focus of investigation into research practices. The following two sections illustrate a practice-based analysis of infrastructure using this case study. The next section centers on the build team and how the practices of pipetting are being reconfigured in light of robotics. The section following the account of pipetting centers on how practices adapt to one another in the case of curation and automated design. Each section centers on mundane routines that use common laboratory objects, and each charts changing procedures, understandings, and interactions and highlights how practices affect one another.

### Reconfiguring Routines: Pipetting

Well plates are abundant in molecular biology. They are sheets of transparent plastic with regularly arranged recesses into which small quantities of reagents, DNA, and cells can be added. Stacks of well plates sit on laboratory benches. Boxes of them sit in store cupboards. Well plates are manufactured by a number of different companies. Plates can come in a range of different volumes, commonly 0.25 ml, 0.5 ml and 1 ml per well. They have a standard sized footprint, originally defined by the Society for Biomolecular Screening (now the Society for Laboratory and Automation), and researchers refer to them by the number of wells on a plate, sometimes with a qualifier for the volume. The common “ninety-six” plate contains recessed wells arranged in a regular eight-by-twelve pattern.

Well plates are indicative and constitutive of the epistemic practices of the bioscience community. For example, researchers typically conduct experiments in triplicate, to account for error and variability, which means the overall number of wells on plates is divisible by three. Experiments tend to be conducted with a control and a number of data points and done in parallel to test different enzymes, strains, or media, for example. A ninety-six plate means that a researcher could, for example, perform four parallel experiments, each with a control and up to seven data points. Well plates, then, can be considered infrastructural objects in microbiology as they have been shaped by, and simultaneously constrain, the performance of contemporary experimental design and practice in terms of volumes, scale, accuracy, and so on, and there is a case for writing a biography of these objects. However, with the advent of automation, it is the routine performances in which well plates are enrolled which can be usefully analyzed as infrastructures.

Micropipetting into well plates is often laborious and repetitive. Adding four different reagents to a ninety-six well plate requires 386 individual aspirates (sample drawn up by suction) and 386 dispenses (ejection of sample) and includes many pipette tip changes (depending on the variability across the wells). There are individual hand micropipettes and multichannel pipettes that can hold eight pipette tips. Micropipetting takes time and concentration but also has a “knack,” meaning some researchers seem to be able to be more consistent than others, meaning their results show less deviation. This variability among researchers is a target for automating the process. Robotics and software innovation are a way for synthetic biologists to move toward their goals of biological science as more standardized, more reproducible, and faster; of realizing biology as technology.

A robot arm can even have up to 96 or 384 micropipettes fixed in the same configuration as a particular well plate, meaning a robot can deliver reagents to all the wells in a plate at once. A common robot arm, and the center has three robots with this configuration, has eight micropipettes that can be moved independently (within certain mechanical parameters) allowing robotic arms to be programmed to aspirate or dispense into different wells, some on different plates, simultaneously. Robotic arms can also be “optimized” to take the “shortest path” to complete all the possible combinations of actions to maximize speed.

The new robotic platforms have “decks” with standardized spaces for well plates (and troughs—which have the same footprint as a well plate but have one large recess in place of twelve, twenty-four, or ninety-six small ones). Robots need to be programmed to pipette at the correct depth, volume, and layout of each plate. This means that programming liquid-handling robots requires a detailed knowledge of both the internal and external dimensions of each well plate. Upon close inspection, it turns out that wells are not a standard size, nor standard shape, and the wells are separated in a particular way depending on their configuration or manufacturer. This means each plate must be carefully measured using a caliper for diameter or width, depth, and configuration. Wells can also have different shaped bottoms—rounded, square, pyramidical, and so on. Furthermore, while the base is a standard footprint, the ways that well plates stack on one another vary. Again, this requires meticulous measurement because if the depth of a plate is out by a millimeter, the error when ten plates are stacked becomes a not-insignificant centimeter. The robot programmer needs to know these shapes in order to ensure all the fluid is pipetted and to prevent “*z*-height crashes” (vertical collisions).

So, whereas a trained bioscientist can take a well plate, and pipette with a relatively high degree of tolerance for minor variation because humans are good at this level of refined motor control, to the robot programmer, each type of plate is strikingly different. One researcher explained that normally, when pipetting manually, he would collect a well plate and a multichannel pipette and start pipetting. But working with robots was different, he exclaimed:

Now, when I see a plate I see more complexity…and they all come with lids! (Direct quotation in field notes, October 5, 2018)

Conventionally, researchers select a well plate depending on the volume indicated by the experimental protocol; the manufacturer of the well plate is, at that point, not important. However, as the researcher explains with respect to robotics, knowledge that did not need to be known (the exact dimensions of wells) or was a form of embodied tacit knowledge (how to dispense into wells with different shaped bottoms) has to be made explicit. The punchline in the field notes above is that the programming researcher has to know well plates in minute detail, even down to the way that a lid rests on the plate, in order to define the well plate for the robot to function correctly.

The researcher now “sees” the well plates differently, as something problematic and complex rather than as simple and ready-to-hand. For robotics to work, some of the labor, design, and practices black-boxed in the well plates have to be de-scribed (Akrich 1992; Ribeiro and Collins 2007) out of infrastructure and re-scripted into infrastructure again by the programmer. This means that well plates in programming, which are routine in manual pipetting practice, expand and unfold for the researcher (Engestrom, Puonti, and Seppanen 2003; Knorr-Cetina 2005). In this case, bringing about a robotic infrastructure in synthetic biology requires new modes of knowledge, technical detail, and accuracy that were not needed for the parallel manual practice, and it is likely these details will once again be hidden in practices of synthetic biology’s epistemic hinterland.

In pipetting, objects that are arguably more mundane and more numerous than well plates are plastic pipette tips. Laboratories can use thousands of tips a day. Not long ago, researchers used to buy bags of sterile tips and place them in holders or racks by hand. Now they can be purchased preracked. When micropipetting manually, a researcher tends to hold the well plate with one hand and micropipette with the other. They press the micropipette nozzle onto a new tip held in the rack, preventing contamination from handling, aspirate their sample, dispense into a given well, and, again to avoid contamination, discard the tip into a waste repository. The racks are in the same layout as well plates so, when micropipetting by hand, scientists can use racks to track which well they are up to. In this way, racks aid the researcher, allowing them to focus on the physical work without losing count, but the rack is more or less invisible; it is the tip that has a “manifest absence” (Law 2004). When operating the robotic machinery, this activity, where the absent tips are an indicator of the researcher’s progress, is abolished. Instead, the ways researchers and robots use the racks and tips become problematic.

The racks do not present the same programming challenge as the well plates as they are manufactured to be compatible with the robots. As already indicated, the robots’ arms do not move like human arms, nor do they have accurate proprioception or feedback from other senses. Unlike a human hand, they do not make microadjustments if a pipette tip is not seated correctly in the holder. The robot arm can descend, meet the edge of a wonky tip, and (provided it does not result in damage) move away without the tip. The robot then senses a missing tip, stops moving, and reports an error. To counteract the possibility of missed tips and a stalled robot, the researchers give the full rack a shake and a tap to ensure that all the tips are “seated” and aligned before they place the rack of tips on the robot deck. They also check the seating visually after they have put the rack on the deck. Thus, reconfiguring pipetting substitutes some of the elements of practice for other actions (Keating, Limoges, and Cambrosio 1999; Ribeiro and Collins 2007).

In the case of pipette tips, the action of using racks and tips changes from being a routine component of research infrastructure to requiring new, albeit minor, problem-solving adjustments. Using robots still requires a “knack,” which researchers attempt to communicate with on-screen messages and to learn by watching one another work the robots. Indeed, the researchers refer to the robots (and computers) as “stupid.” These statements partly serve to remind the researcher that any error is their error, so the robots or computers must be instructed carefully and accurately. This means that as humans creatively adapt to the robots, they preserve elements of infrastructural practice by altering relational repertoires that draw on particular enactments of the differences between humans and machines (see Suchman 2007). The unspoken attitude researchers have toward micropipettes—that they are technical, unthinking objects—is stated explicitly in the case of the robots. “Getting robots to work” indicates a shift in the emotional dimensions of pipetting and, by extension, researchers’ engagements with the experimental practices within which pipetting is embedded.

This account shows that the existing base on which automated liquid handling is built can be understood as composed of as bodily, affective, and routine practice. The existing ways of pipetting, constituted of the necessary knowledge, objects, and procedures (rather than micropipettes and pipette tips) embedded in scientific work, can be thought of as an infrastructure in biological research being displaced by practices of robotics and programming. This means that as the facets of pipetting are disturbed and problematized, actors develop new routines—programming robots; checking and tapping racks; writing operator instructions—that work to reestablish the pipetting as an infrastructural practice. Reconfiguration takes time, trials, and effort. The mundane and embodied processes crucial to the conduct of biology can become unsettled and these configurations, following theories of practice, are what is at stake as practices affect one another. Relations between practices are explored further in the next section.

### Troubling Reproducibility: Curating Frozen Materials

At the end of experiments, when cells containing novel plasmid “assemblies” are produced on a well plate, the researchers typically produce a “stamp,” which is a material copy of the plate. This involves pipetting a sample from each well into the wells of a lower-volume plate (usually using automated liquid handling), sealing the second plate with an aluminum film, and freezing this stamp plate. In this way, duplicates of the experimental materials, usually cultures of cells containing novel DNA plasmids, can be stored in a freezer. If these materials are needed for another experiment, the plate can be thawed and the cells regrown or plasmids replicated. Freezing is therefore an important component of biological research practice because it preserves cells and DNA, meaning that otherwise degradable biological materials are accessible at a future point (see Landecker [2007] for a discussion).

In the center, curating frozen materials has three important functions related to biological experimentation, reproducibility, and credibility. Firstly, “raw” materials for experiments can be stored and retrieved as required for experiments. This short-term storage is usually done using −20 °C freezers. Secondly, longer-term storage can be achieved using −80 °C temperatures. Long-term storage means that experimental teams had a repository of their “raw” experimental materials as well as material outcomes of experiments (e.g., cellular strains containing designed and assembled DNA sequences), which could be accessed at a later date. Thirdly, freezing means other research groups are able to request samples to replicate experiments or, more commonly, adapt and run their own experiments making the capability to locate, retrieve (and send) a requested sample quickly important to the center. Reproducibility and credibility are particularly important, partly because improving reproducibility is a stated general aim of the synthetic biology enterprise (Synthetic Biology Leadership Council 2016) but also because the center began publishing articles and it was conceivable that requests for biological parts and cells might be made.

The research center, with its interdisciplinary team and automations, can “multiplex” experiments, which means that they can run experiments simultaneously, and they can choose to run experiments from more than one project in parallel. In this context of greater experimental “bandwidth,” researchers highlighted the issue of being able to track both *in silico* designs and experimental materials through the center’s design–build–test “pipeline.” A member of the center referred to this problem in terms of linking “theoretical” and “real” entities. The capabilities afforded to biological science by automated design present challenges to established storage and retrieval practices in biology, of which two are detailed and discussed below. These are the challenges of increasing the speed of production of design data and material parts and the historical steps of attempting to standardize the handling of data.

In order to locate and store different biological and chemical materials, the center uses a laboratory data management software called Lab Collector. The application allows the team to create a searchable database that records the storage locations of important resources and experimental materials. The different objects in the database can be linked, which improves the search and retrieval function of curation. For example, linking all the entries of all the materials (DNA primers, bridging oligos, plasmids, barcodes, genes, cell strain) for a particular experimental design means that searching for that design would bring up all the DNA parts and cells relevant to that particular set of experiments. Detailed, linked records mean that, should anyone request samples in the future, all the materials should be easy to identify and retrieve. “Manual curation” is a key part of the current “data-centric biology” where skills in attributing metadata and therefore connecting data across different disciplinary domains are of value in dissemination (Leonelli 2016). In contrast, in the synthetic biology center, it is the skills of connecting the material and the digital, alongside an ability to interlink relevant database objects to one another, that require intimate experimental knowledge and that are crucial to enabling the center’s aim of achieving reproducible science.

In line with a central ethos of synthetic biology, an outcome of applying automated design processes to biological work is an increase in the speed with which researchers can produce information for designing sequences of DNA and experimental combinations. The design team keeps track of designed parts using a coding system and program separate to Lab Collector. Each individually designed part, cell, and plasmid has its own center number. The team also makes use of the online registry of biological parts called the Inventory of Composable Elements (ICE; ICE: https://public-registry.jbei.org/login). So, when the design team creates a given DNA sequence, they also enter the design into ICE, which then generates an ICE number. The center uses this number to track orders from DNA synthesis companies. However, capabilities emerge at different rates (Bowker et al. 2010; Mackenzie 2013), and there is currently a time lag, in the order of several weeks or months, between digitally generating and ordering a new DNA sequence and subsequently having the materials delivered to the laboratory from the synthesis company.

A crucial step for the team, therefore, means ensuring that the physical materials received from a DNA synthesis company match up with the correct ICE numbers and the correct center numbers, meaning that various “theoretical” and “real” parts are linked and correctly catalogued when the orders arrive. This usually involves having a spreadsheet file that lists the center design numbers, the synthesis company number, and the ICE number, then manually sticking labels to the freezer well plates or recording tube location within freezer racks. In addition, as DNA synthesis is a relatively new and unsettled industry, companies can fail or can buy other companies, which can impact their in-house coding mechanisms including customer order histories. This dynamic market increases the importance of accurate center storage and record-keeping.

The second challenge to curation is that the coding systems and digital storage solutions took several iterations to standardize, so team members had to keep track of how newer coding systems related to older coding systems. However, this left myriad interim spreadsheet files in different formats from different center projects detailing the matches between various codes; information infrastructures often require ad hoc fixes (Bowker et al. 2010). The team could enter these codes manually into Lab Collector, but the entries for particular experiments were not all linked to one another. In turn, this generates the laborious technical tasks of matching up all the “legacy” codes and manually linking together the various entries in Lab Collector and then linking these entries to each respective ICE entry to make a large cross-referenced repository. The manual checking process was made more complex by (a) the increase in the number of parts facilitated by automated design and (b) the relative similarity of company codes, center codes, and ICE numbers. There was a sense among some members of the team of being overwhelmed by the amount of data that needed inputting and linking.

In the fourth year of the five-year center, several members of the experimental and technical teams had a series of meetings. The technical team presented an argument that convinced the computer scientists that the troubles of curation and storage could threaten their practices of reproducibility; the hidden technical practices became visible only because of the menace of failure (Shapin 1989). They discussed the issues of inputting data on designs, the legacy data, and recording future deliveries. The team agreed on a strategy that would ultimately (beyond the time frame of the research reported here) automate the input of the legacy spreadsheets as well as automatically link computer-generated DNA designs to future incoming commercial orders. Thus, the infrastructure of curating frozen materials began to be remade with computer science to fit the emerging capabilities of automated design and off-site DNA synthesis.

The team recognized that curating frozen materials currently plays valuable roles in creating the conditions for reproducing biological experiments and achieving research integrity and is, consequently, an important preserver of academic credibility in contemporary bioscience. This raises issues of sharing, access, and compatibility over time, which can be compounded by the temporal unevenness in how infrastructures are established. Automated design of biological material produces unexpected challenges to the infrastructural practices of reproducibility in bioscience, particularly where designs are large, complex, and multipart. The substantive difference between this argument and the case presented here is that, in the synthetic biology center, digital designs and material objects relate to one another such that changing the methodology for creating DNA sequences has a knock-on effect on the capability to curate biological materials. The storage and retrieval of digital data over time is crucial to science. Arguably, focusing on digital infrastructures that make data transportable may overlook the problem that biological materials also have to be made ready for travel. It is useful to conceptualize curating frozen materials as an infrastructural practice in this context since such an analysis highlights multiple ways in which an important element of reproducibility in bioscience, curating frozen materials, is challenged and reconfigured by automated processes.

### Movements, Temporalities, and Ethics

This article raises awareness of considerations for automating academic research. Firstly, implementing automations in robotics and software reconfigures important mundane activities. Routine and established practices, from manual skills like micropipetting to accurate and careful curation, are problematized by the arrival of robotics programming and automated design. Finding ways to accommodate the existing epistemic infrastructure of molecular biology into a new architecture, being created in a zone overlapping academic and commercial interests that value ownership and openness in different ways, takes time and creative responses. Secondly, collaborations can take different forms, and creative epistemic practices can pose new challenges for collaborators where there is little history of prior interaction such as between researchers in one discipline and technical staff in another. This also has potential implications for productivity and realizing faster science. Finally, making a “more reproducible” science is problematic if collaborating disciplines practice reproducibility in different ways. Attention to these infrastructural movements and differences may aid teams to work together to realize the promises of synthetic biology. In other words, considering how hidden research practices are affected, created, or erased by attempts to automate biological research means concentrating on the material and affective routines that already litter epistemic practice.

This article makes several contributions to debates regarding knowledge production infrastructures and infrastructure studies more broadly. To date, where practice is addressed in the infrastructure literature, it tends to be conceived of as “work practices” or as separated from, but linked to, infrastructure (Bowker and Star 1999; Star and Ruhleder 1996, 113). The framework advanced in this paper positions infrastructure as a performed relation between routine practices and formally considers infrastructure as the material procedures and affective repertoires that constitute practice. This means that an infrastructural research practice, such as the hidden activities that achieve some of the core processes of science, can become the focus of creative problem-solving activities, which are employed to stabilize these activities as they are unsettled by new technologies.

Formalizing the concept of infrastructure in this way impacts the popular “inversion” methodology of infrastructure studies that seek to unpack the ways infrastructural objects are produced. This shift foregrounds different materials, knowledges, skills, and aims bound up in routines that are, typically, in the background of knowledge production, yet are fundamental to the processes of science (cf. Shapin 1989). In this way, the above accounts showing how micropipetting and curation can be considered as infrastructural practice demonstrate that as researchers implement new automated developments, routine practices appear to shift out of unproblematic infrastructural roles and move to a realm of creative problem-solving. The example accounts of installations are partial: they offer no end point or ultimate conclusion suggesting that infrastructural practice is an outcome of ongoing performances and further implying research infrastructures can be considered precarious. Therefore, rather than conceiving of installation as a contingent and unidirectional sedimentation of decisions, agreements, and then maintenance, this view shows how infrastructural practices may be embedded in new configurations, which offers more possibilities for describing the biographies and fates of infrastructures.

Placing practice at the center of studies lends processes of creating knowledge production infrastructure even more fluidity than found in previous debates. The “installed base” can be considered, not a static material or convention of logic but as itself constituted of a sea of practice. Indeed, people are “trained to infrastructure” which includes understandings of which components of practice are anchored and which more mobile (cf. Ribes and Polk [2015] for holding objects of research infrastructure in place; Rheinberger [1997] for an account of stabilizing elements to facilitate experimentation). This training is partly what submerges particular practices since those that enact key goals can be the most prosaic. As demonstrated in the above accounts of knowledge production, some scientific intentions remained stable: researchers were not trying to remake the grounds for reproducibility or the conventions of valid knowledge (e.g., triplicate experiments) but were trying to adapt infrastructural practices so that these intentions were insulated or made better by change. Thus, overarching aims of the practices (maintaining epistemological and reproducibility expectations) were preserved through particular adaptations in which elements of material practice (expanding pipetting know-how, automating data linkage) and affective practice (emotional statements about machines, collaborating for reproducibility) shifted. An explicitly practice-based analysis therefore draws more attention to movement, to sinking and surfacing routines, and asks which components of practice are fastened in place and which are unmoored and subject to reconfiguration.

This understanding of infrastructure therefore supplements previous arguments that focus on a “spatial” approach to distribution (Bowker et al. 2010) by showing how dimensions of practice are differentially affected and also builds on the underexplored “when” of infrastructure (Star and Ruhleder 1996) by drawing attention to different temporal aspects of infrastructures. The accounts of micropipetting and curating frozen materials show how practices can gain and lose infrastructural properties over time. Attention to temporality means that, as details and components of practices become problematic, infrastructural routines can be unsettled, expand, and rise up and become the focus of problem-solving. Secondly, even where the goals of infrastructural practices are similar, specific activities happen at different rates in different spaces, and this can lead to difficulties in resolving processes such that they are once again rendered unproblematic. Acknowledging the timings of transformations and movements can give new insights into how different elements of practice are troubled, negotiated, and reorganized; how different practices can become relatively more or less infrastructural; and, crucially, how different practices enroll and organize one another into infrastructural roles.

The notion of infrastructure as emerging from relations between practices therefore changes the ethical implications for analyses. The literature often locates problems of infrastructure with social groups or things (Pinch 2010; Vertesi 2014). Vertesi (2014), for instance, argues that different infrastructures need to be aligned to carry out a particular practice, which leaves the actor or group vulnerable: if they cannot bring about the correct alignment, the actor, as a communicant, loses their voice. Alternatively, it is the fault of infrastructural objects or their design. Shifting to an understanding of infrastructure as practice moves, the ethical questions onto charting which practices are involved and how they affect one another rather than how individuals and groups relate to objects or one another. This means that, in cases such as curating biological parts for reproducibility and credibility, changing epistemic routines elsewhere by using automated experimental design has implications for those related activities.

Automating some components of practices can render other components problematic in surprising ways. The ethical questions in knowledge production then begin to be formulated in terms of which elements of practices are stabilized, such as the teleological orientation and, perhaps more importantly, how activities transform one another into and out of infrastructural roles, shifting action into and out of the epistemic foreground, and leads to questions about what it means to value particular practices in interdisciplinary research and how. This paper suggests that there are possibilities for more studies of infrastructure that place routine practices at the center of analysis. In this approach, new ways of conceptualizing ethical issues and new ways of addressing old problems, such as the “when” of infrastructure, may be found.
